# Parkinson’s Kinetigraph for Wearable Sensor Detection of Clinically Unrecognized Early-Morning Akinesia in Parkinson’s Disease: A Case Report-Based Observation

**DOI:** 10.3390/s24103045

**Published:** 2024-05-11

**Authors:** Karolina Poplawska-Domaszewicz, Naomi Limbachiya, Yue Hui Lau, Kallol Ray Chaudhuri

**Affiliations:** 1 Department of Neurology, Poznan University of Medical Sciences, 60-355 Poznan, Poland; 2 Department of Basic and Clinical Neuroscience, Institute of Psychiatry, Psychology and Neuroscience, King’s College London, London SE5 9RX, UK; naomi.limbachiya@kcl.ac.uk (N.L.); ray.chaudhuri@kcl.ac.uk (K.R.C.); 3 Parkinson’s Foundation Centre of Excellence, King’s College Hospital, Denmark Hill, London SE5 9RS, UK; andrealau38@gmail.com; 4 Division of Neurology, Medical Department, Tengku Ampuan Rahimah Hospital, Klang 41200, Malaysia

**Keywords:** Parkinson’s disease, PKG, early-morning off, early-morning akinesia

## Abstract

Early-morning off periods, causing early-morning akinesia, can lead to significant motor and nonmotor morbidity in levodopa-treated fluctuating Parkinson’s disease (PD) cases. Despite validated bedside scales in clinical practice, such early-morning off periods may remain undetected unless specific wearable technologies, such as the Parkinson’s KinetiGraph™ (PKG) watch, are used. We report five PD cases for whom the PKG detected early-morning off periods that were initially clinically undetected and as such, untreated. These five cases serve as exemplars of this clinical gap in care. Post-PKG assessment, clinicians were alerted and targeted therapies helped abolish the early-morning off periods.

## 1. Introduction

Levodopa (LD) remains the mainstay for treatment of Parkinson’s disease (PD), most effective during the early stages of the disease [1]. However, prolonged LD usage often precipitates motor fluctuations and the emergence of “off-states”, characterized by a diminution, delay, or absence of its therapeutic effects, thereby triggering the re-emergence of motor and nonmotor PD symptoms [1,2,3,4]. Early-morning off period or early-morning akinesia (EMO) is one example of this and afflicts approximately 59.7% of people with PD (PwPD), irrespective of disease stage [2].

EMO is a well-established clinical phenomenon in LD-treated PD, yet it often evades clinical recognition and subsequently remains untreated. The burden of untreated and unrecognized EMO leads to reductions in patient quality of life [2] and increases in caregiver burden [5], with affected individuals reported to experience poorer quality of sleep and more motor and nonmotor symptoms [6]. In an international multicenter study involving 320 patients, EMO was reported in 59.7% (54.6% of males and 68.7% of females), with 88.0% of patients experiencing troublesome EMO characterized by motor and nonmotor symptoms [2]. Nonmotor symptoms included depression, pain, anxiety, and urgency of urination, and in most cases, the symptoms were unrecognized prior to the study and consequently untreated. The scale of this problem is further exemplified in a recent long-term tolerability study of subcutaneous foslevodopa/foscarbidopa, whereby EMO was observed in 77.7% of cases [7]. Despite its significance, research into EMO remains limited, and short clinic visits spaced over extensive intervals complicate and delay accurate screening and diagnosis of EMO.

Recently, the integration of wearable sensor technology during clinic visits has been shown to serve as an objective aid in the clinical assessment of patients. A range of such devices are available, and several have been reviewed by NICE, including Kinesia 360 (Great Lakes Neurotechnologies Inc., Cleveland, OH, USA) and PD monitors (PD Neurotechnology). However, there is a scarcity of data regarding the usefulness of these devices for recording night-time data. The Parkinson’s KinetiGraph™ (also known as the Personal KinetiGraph™ or PKG™, Global Kinetics Corporation, Melbourne, Australia) [8,9] is a wrist-worn sensor, capable of monitoring and generating reports of various PD parameters, including motor fluctuations, immobility, adherence to medication, tremor, dyskinesia, bradykinesia (BK), and immobility [10]. PKG data have been shown to be useful for several aspects of monitoring night-time motor function in PD, the primary focus of our report, as shown by the previous literature [2,3,4]. Furthermore, PKG has been utilized at our center since 2014, supported by a multicenter registry, a good cost–benefit record, and high patient acceptability. It is for these reasons that we used PKG in this report [10].

Despite its potential to guide clinical decisions, no studies have yet explored its effectiveness in monitoring EMO. Therefore, we conducted an audit of our patients with PKG recordings, specifically selecting those with documented EMO parameters, and subsequently performed a retrospective clinical assessment.

## 2. Methods

From our comprehensive dataset of 200 cases (with detailed findings to be reported separately), we present the initial analysis of five cases where PKG monitoring was implemented. Patients were recruited from PKG centers at King’s College Hospital, King’s Parkinson’s Centre of Excellence, and the PKG Polish center at Poznan University, Poland. The integration of the PKG watch into our clinical pathway was prompted by our observations of suboptimal motor control in patients during clinic visits.

We conducted a retrospective examination of clinical records for cases with documented PKG data to assess whether clinicians had identified and addressed episodes of excessive motor fluctuations. Records were independently assessed by two observers (KPD and KRC) for all five cases and graded as positive for EMO based on visual assessment and BK percentile scores in the report (average of seven days) between the 2 AM–6 AM period.

A preliminary assessment of patients’ Parkinson’s disease sleep scale (PDSS) indicated prevalent sleep disturbances (PDSS < 100) and instances of EMO (scores below 6 on PDSS items 12/13/14) across all cases. Notably, none of the patients were undergoing targeted night-time dopaminergic therapies [6]. While our registry encompasses other cases where PKG data supported clinical assessments and guided appropriate interventions, these cases underscore instances where clinical diagnosis overlooked signs evident in PDSS assessments. We selected these cases as exemplars. The combination of PDSS insights and the objective, quantified data provided by the PKG facilitated tailored care strategies for these individuals.

The PKG watch provides objective, continuous, and automated remote (home-based) assessment of motor and night-time symptoms of PD, based on average recording data from a 7-day period. The device is typically worn on the dominant affected wrist of the subject with PD and features a 3-axis accelerometer that records acceleration at a sampling rate of 50 samples per second, ensuring near-continuous recording. The data are captured continuously over a 6–7-day period, and then downloaded, quantified using an established algorithm, and correlated with medication intake timing to generate a detailed report. This report provides mean scores with cut-off parameters for BK, dyskinesia, tremor, immobility at night, and medication adherence. From the PKG records, various patterns of off periods in terms of timing, duration, and severity can be visually recognized and graded. Additionally, periods when the watch is not worn are also recorded.

In the context of this report, the algorithm that identifies BK is of particular importance. BK is recorded as epochs of movements with lower acceleration and amplitude, as well as longer intervals between movement patterns. The device recordings allow for the grading of severity levels for BK, defined based on the average 50th, 75th, and 90th percentiles of BK [8,11]. Additionally, sleep fragmentation and sleep quality data are available by means of 24-h PKG recording [12,13,14].

Furthermore, a clinical assessment of both daytime and nocturnal sleep is feasible based on the percentage time immobile index. This index has been correlated with data from the PDSS [12]. The PDSS is a composite measure of motor and nonmotor aspects of sleep dysfunction in PD and can indirectly indicate the occurrence of EMO based on several items such as early-morning tremor, dystonia, and lack of sleep refreshment.

The PDSS stands out as one of the most rigorously validated scales for assessing sleep in PD, widely adopted since its validation in 2002 [15]. Scores below 100 on the PDSS indicate impaired sleep, with individual symptom impairment reflected by scores below 6. Specifically, a score below 6 on items 12 (early-morning off-related dystonia), 13 (early-morning off-related tremor), and 14 (lack of sleep refreshment upon waking) of the PDSS typically signals significant early-morning akinesia and/or off periods. The PDSS is recommended by the Movement Disorders Society task force for clinical assessment of a range of sleep disorders and severity in PD [16].

This was an audit of clinical cases and, as such, ethics approval was not required. The PKG registry and scale-based assessments (PDSS) were part of the PKG registry (REC reference: 17/LO/1010, IRAS ID: 215965) and a PD nonmotor longitudinal cohort study (NILS) for which ethics approval was in place (REC reference: 10/H0808/141; IRAS ID: 62343).

## 3. Results

**Figure 1:**
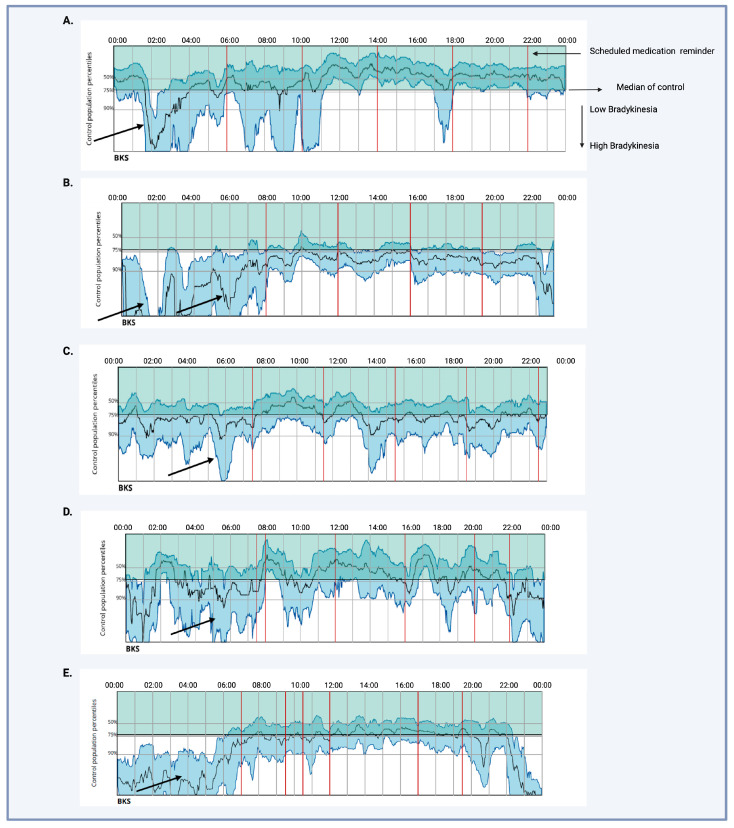
The median seven-day average of BK scores as detected by PKG recording in the five cases described in this paper. Each case is denoted as cases (**A**–**E**), and arrows indicate the visual recognition of early-morning 02–06 AM “dips” or “troughs” in BK corresponding with EMO-related symptoms. Red vertical lines represent the time of day when medication reminders were scheduled. Dyskinesia is represented by green lines, while blue lines denote BK scores. Higher placement on the graph corresponds to a higher dyskinesia score, indicating worsening symptoms. Conversely, lower placement indicates a higher BK score, signaling worsening BK. The horizontal black line represents the median control level, with the time of day indicated above. Created with BioRender.com.

Five patients with PD were identified as having EMO between October 2023 and December 2023. Table 1 summarizes the demographics and relevant PDSS and PKG details for each patient case. We observed varying patterns of severe early-morning BK, including recurrent troughs or persistent BK, typically occurring between 02:00 and 06:00 AM, aligning with patient-reported symptoms and PDSS scores (Figure 1). Notably, EMO went unnoticed in these patients prior to PKG use, despite indications from the PDSS, and as such, no treatment was offered. Following PKG documentation, treatment strategies were implemented, including night-time continuous drug delivery such as transdermal rotigotine patch administration and nocturnal dosing of catechol-o-methyl-transferase inhibitors alongside LD. These interventions proved effective in alleviating early-morning BK post-PKG assessment.

### Case Descriptions

Case 1: This individual is a 71-year-old male who has been diagnosed with PD for the past 18 years. At the time of assessment, he was classified as stage 3 according to the Hoehn and Yahr (H&Y) scale, experiencing optimally on periods, with a total LEDD of 510 mg, managed without advanced infusion therapies. Notably, his BK score was 15.6, which is within normal limits; however, visual analysis revealed a sharp drop in BK scores between 2 and 4 AM (Figure 1A). His sleep was fragmented, with a sleep quality of 61.7% and a sleep fragment duration of 26 min. The PDSS assessment conducted in the clinic showed impairment, with a total score below 83, and items 12, 13, and 14 exhibited scores between 6 and 7.

Case 2: A 59-year-old female has been diagnosed with PD for 5 years. During the assessment, her H&Y scale was 2, with a total LEDD of 350 mg. She was managing her condition without any advanced therapies and her PDSS score was below 85, suggesting sleep impairment as well as evidence of clinical early-morning akinesia and tremor on items 13/14 of the PDSS. Her BK score was 28.5 (normal < 25) and visual analysis of the PKG mean 7-day record showed early-morning steep “dips” in BK scores between 2 and 6 AM, with two “troughs” at 3 and 6 Am (arrowed in Figure 1B).

Case 3: A 62-year-old female has been diagnosed with PD for 11 years. Her H&Y scale was 2 in her recent assessment, and she was treated with a daily LEDD of 820 mg. Symptoms of sleep disruption and EMO were present, with PDSS scores below 83 and an impaired item 14 score. Her overall 7-day BK score was 23.9, within the normal range, but visual assessment of BK records showed BK troughs at 2 and 6 AM (shown by arrows in Figure 1C), consistent with the patient’s history. Sleep quality was poor at 48.3%, with a sleep fragment duration of 24 min. She was not on any targeted night-time therapy.

Case 4: A 77-year-old female has been diagnosed with PD for the past 26 years. Her recent evaluation revealed an H&Y scale of 3, with a total LEDD of 1162 mg, and she was managed on advanced therapy using intrajejunal levodopa infusion. Her BK score was 20.5, with a sleep fragment duration of 28 min and a sleep quality of 79.8. Visual PKG assessment showed frequent troughs in BK between 2 and 6 AM (shown by arrows in Figure 1D). Clinically, she exhibited EMO with an impaired PDSS total score as well as impaired scores on items 13 and 14.

Case 5: This 63-year-old man had a 6-year history of PD and presented with painful off-related dystonia, poor sleep, and restless legs syndrome. He was maintained on an LEDD of 1100 mg/day. The PKG recording showed a median BK score of 23.8, with poor sleep quality of 33 and a sleep fragment duration of 10 min. A visual dip in BK was evident from 2 to 6 AM, when he reported maximum painful dystonia and off symptoms, and his PDSS score was 53 (shown by arrows in Figure 1E).

## 4. Discussion

This short case series of five cases identified from an international PKG case registry dataset highlights the following key points:

Nocturnal or early-morning akinesia and off periods can be detected using the PKG, highlighting its significance as a surrogate technology. Clinical indicators are typically available through the PDSS, where symptoms manifest as a diminished total score and impairments in items 12, 13, and 14, either collectively or individually. It is noteworthy that despite seemingly normal average PKG BK scores over a 7-day remote monitoring period at home, as observed in four out of our five cases, instances of EMO can still occur. Furthermore, the PDSS is unable to predict the precise timing of the EMO periods as is possible with PKG records where the data are logged hourly in the report. Therefore, suggesting a thorough examination of PKG records alongside clinical validation may enhance the detection and accurate assessment of EMO.

These five cases were specifically chosen because, in each instance, examination of PKG data prompted the clinical recognition of EMO periods, which had previously gone unnoticed and untreated. This was in part thought to arise from daytime BK scores falling within the normal range, as presented in four out of five cases, indicating that clinicians primarily focused on daytime PD management. However, these cases highlight that normal daytime motor control does not preclude the presence of EMO periods. Although there may be dispute surrounding PKG data interpretation, in all five cases, targeted night-time dopaminergic treatment was initiated post-PKG assessment, resulting in the alleviation of EMO symptoms. This targeted treatment was initiated following clinicians’ awareness of symptoms visualized through PKG records, despite suggestions from the PDSS that had not been addressed previously.

### 4.1. Wearable Sensor Use

The effectiveness of clinical care is often compounded of incorrect patient self-reporting and poor awareness of physicians, whereby symptoms are often confused and not recognized [17]. Efforts to address this issue have led to the development of EMO screening tools, such as the Time-to-On questionnaire (TOQ) and a seven-question screening tool [3,18]. In clinical practice, most centers of excellence use the patient completed PDSS, which is available globally and effectively signposts symptoms such as tremor or dystonia in the early morning or a lack of sleep refreshment upon waking, specifically in items 12–14. However, such clinical or patient-reported tools have limitations, including reliance on patient memory along with potential recall bias and self-perception bias.

The PKG is easy to use and now has wide validation, with recent endorsement by NICE in the UK [19], as well as a positive cost–benefit analysis suggesting healthcare cost savings [10]. It is increasingly used in clinical practice and provides seven-day monitoring of motor state during the day and night at home, capable of detecting EMO periods. In the present study, all five cases with PKG-documented EMO reported impairment in the PDSS total score, including items 12–14. Moreover, clinical symptoms of early-morning stiffness, difficulty turning in bed, musculoskeletal pain, sweating, and urinary urgency were reported. These observations closely align with previous reports detailing the characteristic features of EMO [2]. However, none of these symptoms were clinically treated, nor were they declared by the patients before the PKG was used to monitor their general motor state. Subsequent targeted therapies with night-time dopamine replacement strategies successfully alleviated the EMO symptoms.

### 4.2. The Role of the Case Reports

These cases therefore highlight the important contribution of wearable sensors such as the PKG in clinical practice, although many neurologists continue to be averse to the use of digital technologies. The issue is even more topical as recent licensing of therapeutics such as subcutaneous foslevodopa/foscarbidopa is specifically aimed toward 24 h treatment and alleviation of early-morning akinesia. The use of wearables such as the PKG would therefore augment such therapies and consequently improve sleep and quality of life for many LD-treated PD patients with EMO periods and akinesia. There are, of course, limitations to these observations and a small number of cases would be cited as one. However, these cases are utilized as exemplars of a clinical problem that may require a large-scale formal observational analysis, which we intend to soon publish with our PKG registry of over 200 records.

## 5. Conclusions

PKG inspection provides an objective indication of EMO episodes, which may otherwise go undetected and consequently untreated with clinical evaluation alone. The PDSS stands as a rigorously validated scale, widely adopted globally. However, it relies on patient input and operates within a visual analogue paradigm. Beyond assessing excessive daytime sleepiness (EMO), the PDSS also encompasses other vital parameters such as sleep quality, refreshment, restless legs syndrome, nocturia, and insomnia. Consequently, the PDSS can aptly serve as a preliminary indicator of EMO, whilst subsequent confirmation and further diagnosis of EMO can then be facilitated through the complementary use of the PKG, particularly in specialized centers. This sequential approach is significant, as evidenced by our cases, wherein the initiation of appropriate treatment followed analysis of the PKG data. While clinically validated scales such as the PDSS highlight EMO periods, our case studies suggest that clinicians’ attention to this issue is improved when night-time sensor data are incorporated. As found here, these data have the potential to change treatment implementation for those with EMO. We conclude that night-time PKG recording is valuable for detecting patterns of early-morning BK, signposting symptomatic and troublesome EMO, and subsequently leading to appropriate treatment for LD-treated PD patients. Integration of the PKG into routine clinical practice may aid clinical evaluations.

## Figures and Tables

**Table 1:** Demographics and clinical overview of five case studies with untreated early-morning off.

	Case 1	Case 2	Case 3	Case 4	Case 5
Gender	M	F	F	F	M
PD duration (years)	18	5	11	26	1
Current age (years)	71	59	62	77	82
Hoehn and Yahr stage (motor)	3	2	2	3	4
LEDD (mg)	510	350	820	1162	600
Oral or infusion therapy	Oral	Oral	Oral	LCIG	Oral
PDSS score (abnormal < 100)	83	83	85	<100	53
Median BK score	50.6	28.5	23.9	20.5	23.8
Sleep quality (PKG)	61.7	NA	48.3	79.8	33
Severe visual “dips” in BK line between 2 AM and 6 AM	Yes	Yes	Yes	Yes	Yes

PD, Parkinson’s disease; M, Male; F, Female; LEDD, levodopa equivalent daily dose; LCIG, levodopa-carbidopa intestinal gel infusion; PDSS, Parkinson’s disease sleep scale; BK, bradykinesia.

## Data Availability

Data are contained within the article.

## References

[1] Kwon D.K., Kwatra M., Wang J., Ko H.S. (2022). Levodopa-Induced Dyskinesia in Parkinson’s Disease: Pathogenesis and Emerging Treatment Strategies. Cells.

[2] Rizos A., Martinez-Martin P., Odin P., Antonini A., Kessel B., Kozul T.K., Todorova A., Douiri A., Martin A., Stocchi F. (2014). Characterizing motor and non-motor aspects of early-morning off periods in Parkinson’s disease: An international multicenter study. Park. Relat. Disord..

[3] Stocchi F., Coletti C., Bonassi S., Radicati F.G., Vacca L. (2018). Early-morning OFF and levodopa dose failures in patients with Parkinson’s disease attending a routine clinical appointment using Time-to-ON Questionnaire. Eur. J. Neurol..

[4] Suzuki K., Fujita H., Okamura M., Kobayashi S., Hirata K. (2020). Does good sleep reduce early-morning off periods in patients with Parkinson’s disease?. Sleep.

[5] Onozawa R., Tsugawa J., Tsuboi Y., Fukae J., Mishima T., Fujioka S. (2016). The impact of early morning off in Parkinson’s disease on patient quality of life and caregiver burden. J. Neurol. Sci..

[6] Martinez-Martin P., Visser M., Rodriguez-Blazquez C., Marinus J., Chaudhuri K.R., van Hilten J.J. (2008). SCOPA-sleep and PDSS: Two scales for assessment of sleep disorder in Parkinson’s disease. MovMov. Disord..

[7] AldAldred J., Freire-Alvarez E., Amelin A.V., Antonini A., Bergmans B., Bergquist F., Bouchard M., Budur K., Carroll C., Chaudhuri K.R. (2023). Continuous Subcutaneous Foslevodopa/Foscarbidopa in Parkinson’s Disease: Safety and Efficacy Results From a 12-Month, Single-Arm, Open-Label, Phase 3 Study. Neurol. Ther..

[8] Griffiths R.I., Kotschet K., Arfon S., Xu Z.M., Johnson W., Drago J., Evans A., Kempster P., Raghav S., Horne M.K. (2012). Automated assessment of bradykinesia and dyskinesia in Parkinson’s disease. J. Park. Dis..

[9] Khodakarami H., Farzanehfar P., Horne M. (2019). The Use of Data from the Parkinson’s KinetiGraph to Identify Potential Candidates for Device Assisted Therapies. Sensors.

[10] Chaudhuri K.R., Hand A., Obam F., Belsey J. (2022). Cost-effectiveness analysis of the Parkinson’s KinetiGraph and clinical assessment in the management of Parkinson’s disease. J. Med. Econ..

[11] Carroll C., Kobylecki C., Silverdale M., Thomas C. (2019). Impact of Quantitative Assessment of Parkinson’s Disease-Associated Symptoms Using Wearable Technology on Treatment Decisions. J. Park. Dis..

[12] Klingelhoefer L., Rizos A., Sauerbier A., McGregor S., Martinez-Martin P., Reichmann H., Horne M., Chaudhuri K.R. (2016). Night-time sleep in Parkinson’s disease—The potential use of Parkinson’s KinetiGraph: A prospective comparative study. Eur. J. Neurol..

[13] Kotschet K., Johnson W., McGregor S., Kettlewell J., Kyoong A., O’Driscoll D., Turton A., Griffiths R., Horne M. (2014). Daytime sleep in Parkinson’s disease measured by episodes of immobility. Park. Relat. Disord..

[14] McGregor S., Churchward P., Soja K., O’driscoll D., Braybrook M., Khodakarami H., Evans A., Farzanehfar P., Hamilton G., Horne M. (2018). The use of accelerometry as a tool to measure disturbed nocturnal sleep in Parkinson’s disease. NPJ Park. Dis..

[15] Chaudhuri K.R., Pal S., DiMarco A., Whately-Smith C., Bridgman K., Mathew R., Pezzela F.R., Forbes A., Högl B., Trenkwalder C. (2002). The Parkinson’s disease sleep scale: A new instrument for assessing sleep and nocturnal disability in Parkinson’s disease. J. Neurol. Neurosurg. Psychiatry.

[16] Högl B., Arnulf I., Comella C., Ferreira J., Iranzo A., Tilley B., Trenkwalder C., Poewe W., Rascol O., Sampaio C. (2010). Scales to assess sleep impairment in Parkinson’s disease: Critique and recommendations. Mov. Disord..

[17] Braybrook M., O’connor S., Churchward P., Perera T., Farzanehfar P., Horne M. (2016). An Ambulatory Tremor Score for Parkinson’s Disease. J. Park. Dis..

[18] Han C., Mao W., An J., Jiao L., Chan P., on behalf of the China Early Morning Off (EMO) Study Group (2020). Early morning off in patients with Parkinson’s disease: A Chinese nationwide study and a 7-question screening scale. Transl. Neurodegener..

[19] National Institute for Health and Care Excellence (2018). Devices for Remote Monitoring of Parkinson’s Disease. Diagnosis and Management of Parkinson’s Disease: Summary of Updated NICE Guidance, NICE. www.nice.org.uk/guidance/dg51.

